# The relationship between vitamin D and insulin resistance before delivery in advanced maternal age

**DOI:** 10.1186/s12958-019-0555-y

**Published:** 2019-12-18

**Authors:** Beibei Dong, Mengmeng Zhi, Manman Han, Hao Lin, Hong Yu, Ling Li

**Affiliations:** 1 Department of Endocrinology, Zhong Da Hospital, School of Medicine, Southeast University, Nanjing, 210009 Jiangsu Province China; 2 Pancreatic Research Institute, Southeast University, Nanjing, China; 3 Department of Clinical Science and Research, Zhong Da Hospital, School of Medicine, Southeast University, Nanjing, China; 4 Department of Obstetrics and Gynecology, Zhong Da Hospital, School of Medicine, Southeast University, Nanjing, 210009 Jiangsu Province China

**Keywords:** Vitamin D, 25(OH) D, Insulin resistance, Advanced maternal age

## Abstract

**Background:**

With the widely implementation of universal two-child policy, the number of pregnant women in advanced maternal age (AMA) will increase gradually. We aimed to assess the association of vitamin D levels and insulin resistance (IR) during the late pregnancy in AMA.

**Methods:**

A total of 80 pregnant women were consecutively enrolled in the cross-sectional study before delivery from the August 2016 to June 2017 at the department of gynecology and obstetrics in the hospital of ZhongDa, affiliated to Southeast University. At delivery, serum 25(OH) D and metabolism parameters including glucose and lipid levels were measured. IR was calculated by the method of homeostasis model assessment 2(HOMA2).

**Results:**

Pregnant women in AMA with vitamin D deficiency have higher fasting insulin (14.70(8.76–34.65) and 10.89(7.15–16.12), respectively, *P* = 0.031) and HOMA-IR indices (1.78(1.07–4.14) and 1.30(0.83–1.89), respectively, *P* = 0.024) than those with vitamin D non-deficiency. Serum 25(OH) D levels were inversely associated with HOMA-IR indices (r = − 0.25, *P* = 0.025). In multivariable analysis for adjusting confounder factors, vitamin D non-deficiency was also negatively correlated with HOMA-IR compared to vitamin D deficiency (β = − 1.289, *P* = 0.026).

**Conclusions:**

Taken together, our findings suggest that serum 25(OH) D levels were inversely associated with HOMA-IR in AMA. Furthermore, pregnant women in AMA with vitamin D deficiency might have higher HOMA-IR levels than those with vitamin D non-deficiency.

**Trial registration:**

Chinese Clinical Trial Registry (No. ChiCTR-RRC-16008714). retrospectively registered.

## Introduction

In November 2013, the one child policy in China was changed to two child policy that only couple from single-child family can have a second child [1]. In October 2015, the two-child policy was further transformed into universal two child policy and all couples were permitted to have two children [2]. After the implementation of universal two child policy, many older couples already had a child begin to plan for a second child [3]. The Chinese government estimated that 60% of women who benefited from the transformation into universal two child policy are older than 35 years, called as advanced maternal age (AMA) [2].

Several researches have demonstrated that AMA is a risk factor of adverse perinatal outcomes including gestational diabetes (GDM), pre-eclampsia, fetal anomaly and preterm delivery [4, 5]. Among all obstetric complications, a rising prevalence of GDM, characterized by insulin resistance (IR) accompanied with a failure of islet β cells to compensate for it, has been reported over years, reaching 10–15% in the world [6]. Furthermore, the research has demonstrated the pregnancy is a diabetogenic state since the steroid hormones increase and IR occur in peripheral tissues, as well as inflammatory cytokines secreted from adipose tissue and placenta, can contribute to IR and pathogens of GDM [7]. The physiologic condition may be exacerbated by well-established risk factor for GDM, including AMA and overweight and obesity before pregnancy [8].

For decades, vitamin D emerged as a controversial nutrients and pro-hormones. The classical action of vitamin D is the modulation of bone and mineral metabolism [9]. However, increasing evidences suggested several extra skeletal action of vitamin D referred to some chronic conditions including cardiovascular diseases [10], obesity [11], metabolic syndrome [12], some kinds of cancer [13, 14] and autoimmune disease [15]. However, there were few studies focused on AMA to explore the state of vitamin D levels before delivery and investigate the association between the level of vitamin D and IR. Thus, this study was aimed to detect the level of vitamin D before delivery and further demonstrate the relationship between vitamin D and IR in AMA.

## Participants and methods

### Study design

The pregnant women were consecutively recruited before delivery from the August 2016 to June 2017 at the department of gynecology and obstetrics in the ZhongDa hospital, affiliated to Southeast University. The inclusion criteria included maternal age more than and equal to 35 years and singleton pregnancy. The exclusion criterion mainly contained maternal age less than 35 years; diabetes and cardiovascular diseases before pregnancy; severe hepatic and renal disorders and autoimmune diseases prior to pregnancy. Written informed consent was obtained from the pregnant women participated in the study. Besides, this study was approved by the ethics committee for human research.

We collected the demography information including maternal age, pre-pregnancy body mass, age at menarche, the history of pregnancy and delivery, the family history of diabetes and hypertension. The blood sample was attained after fasted at least 8 h when the participant registered in the hospital before delivery. Then the blood sample was centrifuged at 3000 rpm for 10 min. Subsequently, the serum was separated and glucose and lipid metabolism parameters including triglyceride (TG), total cholesterol (TC), and low- and high-density lipoprotein cholesterol (LDL-c, HDL-c) were measured immediately. Serum insulin and 25(OH) D concentrations were measured using ectrochemiluminescence immunoassay (ECLI) in the Department of Clinical Laboratory, Zhong Da hospital affiliated Southeast University. According to standard classification typically used, serum concentration of 25(OH) D was stratified into vitamin D deficiency (< 20 ng/mL) and vitamin D non-deficiency (≥ 20 ng/mL) [16]. Homeostasis model assessment-insulin resistance indices (HOMA-IR) were calculated by update HOMA2 method (based on computer model and provided by the University of Oxford Diabetes Trial Unit) [17].

### Statistical analysis

Data was presented as mean ± standard deviation if it followed the normal distribution. If followed skew distribution, then it will be represented as median and interquartile range. Moreover, categorical variables were depicted as count and percentage. Student *t* test was conducted in continues variables with normal distribution for group comparison and Mann Whitney *U* test was used for continuous variables with skew distribution. Correlation analysis between the serum vitamin D concentration and HOMA-IR was performed by using Spearman correlation analysis. Multivariable analysis was conducted by multivariable linear regression using Enter variables including maternal age, education, pre-pregnancy body mass index, age at menarche, numbers of parity and abortion, glucose and lipid metabolism parameters. All statistical analyses were implemented by SPSS version 23.0. Two-sided *p* values < 0.05 were considered for statistical significance.

## Results

### The baseline characteristic of the study participants

According to the inclusion and exclusion criteria, 80 pregnant women with AMA were included in the study. Then, pregnant women were stratified into vitamin D deficiency and vitamin D non-deficiency on the basis of the level of serum 25(OH) D in the late pregnancy. As shown in the Table 1, pregnant women in the two groups were 36 years in average and were 14 years with the starting of menarche. Women with vitamin D deficiency were mostly lower level of education than those with vitamin D non-deficiency (14 years and 16 years, respectively). However, there was not statistical difference between the two groups. Besides, there were no significant difference among the number of parity and abortion and pre-pregnancy body mass index in the two groups.

**Table 1:** Baseline characteristics of the study population

Characteristics	Vitamin D deficiency (*n* = 40)	Vitamin D Non-deficiency (*n* = 40)	*P*-value
Age (years)	36(35–38)	36(35–39)	0.856
Education (years) −12 12–16 16-	14(12–16) 11(27.5) 25(62.5) 4(10)	16(14–16) 6(15) 30(75) 4(10)	0.080
pre-pregnancy body mass index (kg/m^2^)	23.18 ± 2.56	22.96 ± 2.50	0.689
Age at menarche (years)	14(13–14)	14(13–14)	0.758
Parity (numbers) > 2	3(2–4) 22(55)	3(2–4) 22(55)	0.782
Abortion (numbers)	1(1–2)	1(0–2)	0.491

Data are presented as n (%), mean ± SD or median (interquartile range) as appropriate

### Clinical characteristic of participants in the two groups

**Fig. 1:**
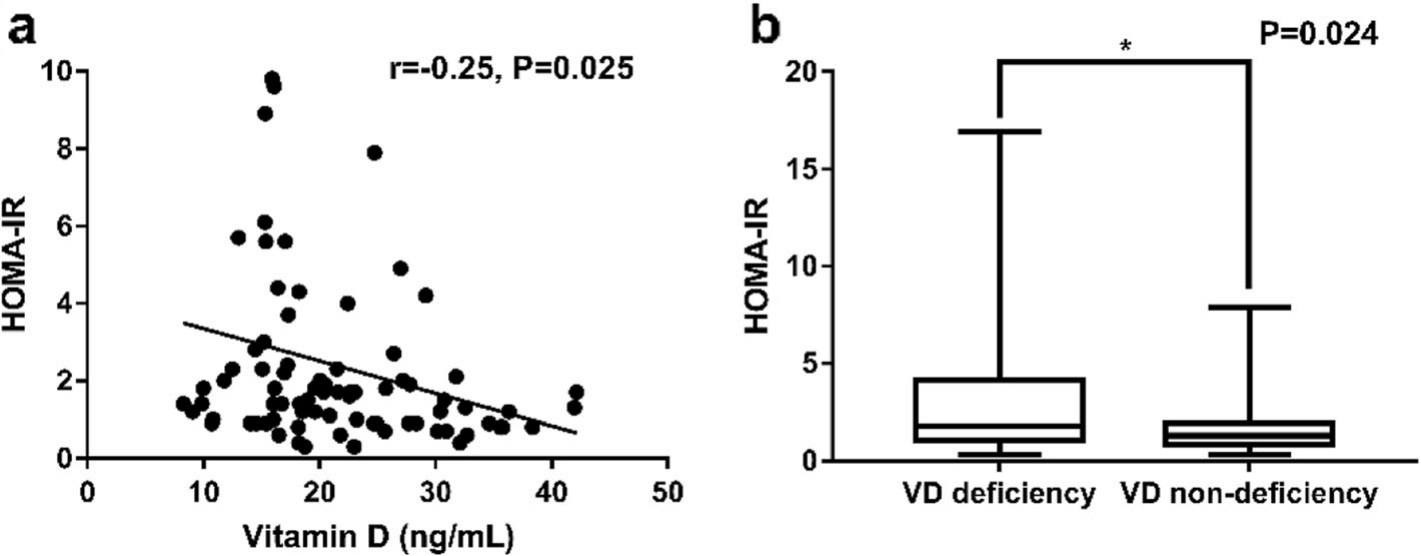
The correlation between serum concentration of Vitamin D and HOMA-IR in the late pregnancy (**a**). Comparison of HOMA-IR index in Vitamin D deficiency and Vitamin D non-deficiency (**b**). * Statistically significant *P*-value < 0.05

Pregnant women in the study delivered at the 39 weeks in average. There was no significant difference in the fasting blood glucose and HOMA-β between the two groups. Moreover, the difference in the serum level of lipid between the two groups was not significant. The fasting blood insulin (14.70(8.76–34.65) and 10.89(7.15–16.12), respectively, *P* = 0.031, Table 2) and HOMA-IR (1.78(1.07–4.14) and 1.30(0.83–1.89), respectively, *P* = 0.024, Fig. 1b) were higher in the vitamin D deficiency group than those in the vitamin D non-deficiency group. The serum level of 25(OH) D was negatively associated with HOMA-IR in all pregnant women (*r* = − 0.25, *P* = 0.025, Fig. 1a). Similarly, HOMA-s% in the vitamin D deficiency group was lower than it in the vitamin D non-deficiency group (0.56(0.24–0.94) and 0.77(0.53–1.20), respectively, *P* = 0.024).

**Table 2:** Clinical Characteristics of participants in the two groups

Characteristics	Vitamin D deficiency (*n* = 40)	Vitamin D Non-deficiency (*n* = 40)	*P*-value
Gestational weeks (week)	39(38.25–39.75)	39(38–39)	0.237
Fasting blood glucose (mmol/L)	4.50(4.20–4.97)	4.35(4.06–4.75)	0.142
Fasting insulin (mIU/mL)	14.70(8.76–34.65)	10.89(7.15–16.12)	0.031*
HOMA-IR	1.78(1.07–4.14)	1.30(0.83–1.89)	0.024*
HOMA-β	1.72(1.36–2.40)	1.61(1.22–1.98)	0.172
HOMA-s%	0.56(0.24–0.94)	0.77(0.53–1.20)	0.024*
TG (mmol/L)	3.81(3.11–5.44)	3.80(2.92–4.99)	0.690
TC (mmol/L)	6.40 ± 1.27	6.20 ± 1.08	0.452
LDL-c (mmol/L)	3.21 ± 0.93	3.07 ± 0.72	0.551
HDL-c (mmol/L)	1.96 ± 0.33	2.01 ± 0.38	0.442
Vitamin D (ng/mL)	15.95(14.15–17.30)	27.08(22.73–32.01)	< 0.001*

Data are presented as n (%), mean ± SD or median (interquartile range) as appropriate

*Abbreviations*: *HOMA-IR* HOMA-insulin resistance, *HOMA-β* HOMA beta-cell function, *HOMA-s%* HOMA-insulin sensitivity, *TG* Triglyceride, *TC* Total cholesterol, *LDL-c* Low density lipoprotein-cholesterol, *HDL-c* High density lipoprotein-cholesterol

### The multivariable analysis among clinical parameters and the level of HOMA-IR

Further multivariable analysis including confounding factors (age, education, pre-pregnancy body mass index, age at menarche, parity, abortion, TG, TC, LDL-c, HDL-c), age at menarche and 25(OH) D levels were significant associated with the level of HOMA-IR. Additionally, women with vitamin D non-deficiency were negatively related with the level of HOMA-IR compared to those with vitamin D deficiency (β = − 1.289, *P* = 0.026, Table 3). Age at menarche was also negatively associated with the level of HOMA-IR (β = − 0.490, *P* = 0.039, Table 3).

**Table 3:** The multivariable analysis among clinical parameters and the level of HOMA-IR

Variables	Unstandardized coefficients β	SE	Standardized coefficients β	t	*P*-value	95 CI % for β
Age at menarche (years)	−0.490	0.233	−0.259	−2.100	0.039*	−0.955 to − 0.024
Vitamin D concentration						
Deficiency	1	1	1	1	1	1
Non-deficiency	−1.289	0.568	−0.245	−2.271	0.026*	−2.422 to −0.156
Constant	15.456	6.698	–	2.308	0.024*	2.091 to 28.821

Data were analyzed by multivariable linear regression using Enter variables. (inclusion variables including Age; Education; Pre-pregnancy body mass index; Age at menarche; Parity; Abortion; TG; TC; LDL-c; HDL-c and Vitamin D concentrations)

*Abbreviation*: *TG* Triglyceride, *TC* Total cholesterol, *LDL-c* Low density lipoprotein-cholesterol, *HDL-c* High density lipoprotein-cholesterol. Vitamin D concentration was stratified into Deficiency (< 20 ng/mL) group and Non-deficiency (≥20 ng/mL) group

*Statistically significant *P*-value < 0.05

## Discussion

This study has demonstrated that the levels of serum 25(OH) D levels in the late pregnancy were negatively associated with IR in AMA (r = − 0.25, *P* = 0.025, Fig. 1a). Furthermore, women with vitamin D deficiency have higher level of fasting blood insulin (14.70(8.76–34.65) and 10.89(7.15–16.12), respectively, *P* = 0.031, Table 2) and HOMA-IR (1.78(1.07–4.14) and 1.30(0.83–1.89), respectively, *P* = 0.024, Fig. 1b) compared to those with vitamin D non-deficiency. After adjusted confounder factors, women with vitamin D non-deficiency were negatively related to IR in comparison to those with vitamin D deficiency (β = − 1.289, *P* = 0.026, Table 3).

In the current study, women with vitamin D deficiency have higher fasting insulin concentration than those with vitamin D non-deficiency. However, there were no significant difference in fasting glucose concentration and lipid level between the two groups. Similarly, several studies have demonstrated that vitamin D was associated with metabolic syndrome including IR [18–20] and obesity [21]. Lu L et al. suggested that there was significant inverse association of 25(OH) D with fasting insulin and HOMA-IR in overweight and obese but not in normal-weight subjects in China (*P* = 0.0363 and *P* = 0.0187, respectively) [22]. Moreover, Chinese individuals with vitamin D deficiency have higher fasting insulin and HOMA-IR compared to those with vitamin D non-deficiency [19]. Furthermore, there were researches identified that pregnant women with low vitamin D in early pregnancy had higher HOMA-IR indices at 28 weeks(r = − 0.32, *P* = 0.02) [23], but not associated with the risk of GDM [24]. Taken together, previous studies have observed the relationship between vitamin D and IR in adults and those association needed further examination were still conflict. Our study was the first to demonstrate that serum 25(OH) D levels in the late pregnancy was statistically associated with fasting blood insulin and HOMA-IR, but not with fasting blood glucose and lipid metabolism in AMA, a worthy more attention population in China.

At the same time, after adjusted some related risk factors of IR and GDM, serum 25(OH) D concentrations in vitamin D non-deficiency group were negatively correlated with the HOMA-IR levels compared to those in vitamin D deficiency group (β = − 1.289, *P* = 0.026, Table 3). As Xiao Y et al. demonstrated that serum 25(OH) D concentrations were significant inversely associated with metabolic covariates including fasting insulin and HOMA-IR, after adjusted for age, sex and BMI (β = − 0.39, *P* < 0.0001 and β = − 1.49, P < 0.0001, respectively) [20]. However, there was another study demonstrated that among males, 25(OH) D was associated with HOMA-IR (β = − 0.011, *P* = 0.004) after adjustment for BMI, but not women [25]. As for the sex-specific relationship, some potential reasons could be that middle-aged males have been shown to have a higher risk of incident metabolic compared with middle-aged women [26, 27]. Whereas, pregnancy was a physiologic condition with gradually increase of insulin concentration especially in the late pregnancy [7]. Therefore, if indeed low vitamin D levels were associated with IR in early pregnancy, there might be a stronger relationship between vitamin D and IR in late pregnancy.

IR is considered as a physiologic condition during the pregnancy and has also be implicated as main characteristic of GDM [28]. In the present study, the levels of IR determined by HOMA-IR were significant higher in vitamin D deficient subjects compared to those in vitamin D non-deficiency, which might be explained by vitamin D involved in glucose metabolism contributed to facilitate the secretion and action of insulin [29, 30].

The strengths of our study were the first to explore the relationship between vitamin D levels and IR before delivery in AMA. Several studies have demonstrated the relationship between vitamin D and diabetes [20, 29], even GDM [24] in pregnant women with common maternal age. Whereas, few studies focus on the association between vitamin D and IR in AMA.

Although those strengths, there are still some limitations of this study needed to be considered. Those pregnant women were recruited from a single hospital. Besides, multivariable analysis was unable to adjust for outdoor activity and lifestyle difference because those were not collected in our study.

### Implications for practice

This study demonstrated the relationship between 25 (OH) D and IR during the late pregnancy in AMA. Whereas this association is based on the background of this study, any causal interpretation of those relationships is not exact and restricted. Thus, well-designed and intervention studies are required to varify whether the relationship between 25 (OH) D and IR during the late pregnancy in AMA from this study are generalizable to all pregnant women. Besides, this study also demonstrated that it’s necessary to strengthen attention to the AMA for reducing the long-term health implication of higher IR before delivery influenced maternal and fetal health. Therefore, establish potent strategies for prevention of vitamin D deficiency in the third trimester of pregnancy may be far-reaching benefits. Obstetricians should pay more attention to provide guidance in monitoring the level of vitamin D in the third trimester of pregnancy and more intervention studies are requested to confirm whether supplement of vitamin D will lead to decreased IR during the late pregnancy.

## Conclusions

In brief, the study was the first to highlight the association between vitamin D and IR before delivery in AMA. Pregnant women in AMA with vitamin D deficiency might have higher HOMA-IR levels than those with vitamin D non-deficiency.

## Data Availability

The datasets used and/or analysed during the current study are available from the corresponding author on reasonable request.

## Abbreviations

AMA: Advanced maternal age
ECLI: Ectrochemiluminescence immunoassay
GDM: Gestational diabetes
HDL-c: High-density lipoprotein cholesterol
HOMA2: Homeostasis model assessment 2
HOMA-IR: Homeostasis model assessment-insulin resistance indices
IR: Insulin resistance
LDL-c: Low-density lipoprotein cholesterol
TC: Total cholesterol
TG: Triglyceride
